# Feasibility of direct brain ^18^F-fluorodeoxyglucose-positron emission tomography attenuation and high-resolution correction methods using deep learning

**DOI:** 10.22038/AOJNMB.2024.74875.1522

**Published:** 2024

**Authors:** Tomohiro Ueda, Kosuke Yamashita, Retsu Kawazoe, Yuta Sayawaki, Yoshiki Morisawa, Ryosuke Kamezaki, Ryuji Ikeda, Shinya Shiraishi, Yoshikazu Uchiyama, Shigeki Ito

**Affiliations:** 1 Graduate School of Health Sciences, Kumamoto University, Japan; 2 Department of Central Radiology Kumamoto University Hospital, Japan; 3 Department of Diagnostic Radiology, Faculty of Life Sciences,Kumamoto University, Japan; 4 Department of Information and Communication Technology, Faculty of Engineering, University of Miyazaki, Japan; 5 Department of Medical Radiation Sciences, Faculty of Life Sciences, Kumamoto University, Japan

**Keywords:** Brain PET, Attenuation correction, High-resolution correction DCNN

## Abstract

**Objective(s):**

To develop the following three attenuation correction (AC) methods for brain ^18^F-fluorodeoxyglucose-positron emission tomography (PET), using deep learning, and to ascertain their precision levels: (i) indirect method; (ii) direct method; and (iii) direct and high-resolution correction (direct+HRC) method.

**Methods:**

We included 53 patients who underwent cranial magnetic resonance imaging (MRI) and computed tomography (CT) and 27 patients who underwent cranial MRI, CT, and PET. After fusion of the magnetic resonance, CT, and PET images, resampling was performed to standardize the field of view and matrix size and prepare the data set. In the indirect method, synthetic CT (SCT) images were generated, whereas in the direct and direct+HRC methods, a U-net structure was used to generate AC images. In the indirect method, attenuation correction was performed using SCT images generated from MRI findings using U-net instead of CT images. In the direct and direct+HRC methods, AC images were generated directly from non-AC images using U-net, followed by image evaluation. The precision levels of AC images generated using the indirect and direct methods were compared based on the normalized mean squared error (NMSE) and structural similarity (SSIM).

**Results:**

Visual inspection revealed no difference between the AC images prepared using CT-based attenuation correction and those prepared using the three methods. The NMSE increased in the order indirect, direct, and direct+HRC methods, with values of 0.281×10^-3^, 4.62×10^-3^, and 12.7×10^-3^, respectively. Moreover, the SSIM of the direct+HRC method was 0.975.

**Conclusion:**

The direct+HRC method enables accurate attenuation without CT exposure and high-resolution correction without dedicated correction programs.

## Introduction

 The techniques for quantitative evaluation of glucose metabolism include use of the standardized uptake value and compartment-model analysis (1). In the former, glucose accumulation in the tissues is quantified in relation to the dose administered, and the radio-activity dose is calibrated for the image count, giving a ratio that can be corrected based on the body weight. The latter is a glucose metabolism quantification method in which a two-tissue compartment model, including three types of rate constants, is applied to ^18^F-fluorodeoxy-glucose (^18^F-FDG), a non- diffusible tracer (2,3).

 Factors related to the quantifiable parameters of positron emission tomography (PET) include the random coincidence coefficient, scattering coincidence coefficient, γ-ray attenuation, spatial resolution, correspondence between PET and computed tomography (CT) images, and correction of radioactivity based on PET values (4). Among these, the factor with the greatest effect on quantitative evaluations is γ-ray attenuation. Thus, attenuation correction is essential for quantitative evaluation (5).

 CT-based attenuation correction (CTAC) is widely used for PET attenuation correction using a PET/CT device (6, 7). In CTAC, the attenuation map obtained by CT is converted to an attenuation map obtained with 511-keV γ-rays. The image is projected, and the attenuation correction coefficient is obtained. Including the attenuation coefficient for the detection probability in the calculation formula for the successive approximation method, reconstruction and simultaneous correction can be achieved (6, 7). This method can generate low-noise attenuation correction data in a short time and simultaneously generate morphological images. However, the CTAC method has disadvantages, such as incorrect fusion due to respiratory movement and additional radiation exposure due to CT.

 Magnetic resonance imaging (MRI) is routinely used for diagnostic imaging because, in comparison with CT, it generates more information and reduces patient exposure, with fewer artifacts and better contrast. Therefore, generation of synthetic CT (SCT) images directly from MRI can enable attenuation correction by the CTAC method without the need for CT devices or radiation exposure.

 However, certain challenges are associated with predicting CT images from MRI findings. The MRI signal intensity does not directly represent electron density, and bone signals cannot be obtained through MRI sequencing at present (7). Deep learning, which generates CT images from MRI images, may help overcome these challenges. Deep convolutional neural networks (DCNNs) are based on the most firmly established algorithms and have also been reported in the field of medical imaging (8, 9).

 Indirect methods has been proposed, in which deep learning can be used to perform CTAC with SCT images predicted from MRI images instead of CT images (10). And direct methods without SCT generation from MRI images also proposed (11-13).

 Hashimoto et al. have reported a highly accurate attenuation correction in brain 18FDG-PET images using DCNN-generated CT images (14). The methods for directly generating CTAC-PET images from non CTAC-PET images using DCNN was reported, and the generated CTAC-PET images had high similarity to the original ones (11, 12). Therefore, although accurate attenuation correction is possible using DCNN, there is a limit to conventional PET resolution in depicting minute structures of the brain. The high resolution of the brain ^18^FDG-PET image helps understand the brain structure in more detail (11, 12). Prior learning through images with high resolution correction (HRC) using DCNN performed in addition to attenuation correction can enable simultaneous attenuation correction and HRC, resulting in higher-quality brain ^18^FDG-PET images. Additionally, it is expected to be highly useful in clinical practice due to its ability to provide accurate correction without exposing patients to CT radiation. This method also provides detailed information about the brain through resolution correction. 

 Therefore, the DCNN based AC with HRC method appears to be a reliable approach, delivering image quality comparable to traditional methods, while also offering additional advantages, particularly in terms of resolution correction for detailed brain structure information.

 We aimed to develop the following three methods for brain ^18^F-FDG-PET, using deep learning, and to ascertain their precision levels: (i) indirect method; (ii) direct method; and (iii) direct+HRC method, with direct AC and HRC performed simultaneously by DCNN.

## Methods

***Ethics statements***

 This study was approved by the Ethics Committee of Medicine at the Kumamoto University for Human Studies (Protocol Number. Advanced 1852, 10/04/2022), and written informed consent was obtained from all patients before the study began. All image data were handled anonymously, and the study was conducted in accordance with the Declaration of Helsinki and the regulations of each institution’s ethics board. This study was conducted following strengthening the Reporting of Observational Studies in Epidemiology (STROBE) guidelines.

***Participants***

 For the indirect and direct methods, the images of the 53 patients were used for the study. Twenty of these patients (12 men: mean age, 77.0 years; range, 63–84 years; and 8 women: mean age, 77.3 years; range, 59–89 years) who underwent both head MRI and CT examinations from February 2020 to August 2021 at Kumamoto University Hospital and 33 cases obtained from the Cancer Imaging Archive (15) (19 men: mean age, 57.7 years; range, 30–70 years; and 14 women: mean age, 65.6 years; range, 48–78 years) were included. To enhance the accuracy of SCT generation, additional image sets were added from the database (15). 

 For the participants included from the Cancer Imaging Archive, the images were obtained at different institutions and, therefore, the matrix size, pixel size, and device used differed between them. The CT tube voltage was 120 or 140 kV, T1-weighted images were used for MRI, and contrast-enhanced imaging was not used for either MRI or CT. The quality of the generated SCT remains consistent regardless of the input MRI sequence (16). Therefore, the T1 weighted images (T1WI) were selected for the MR images in this study.

 For the direct+HRC method, the images of 14 patients who underwent all three imaging modalities, including head MRI and brain PET/CT with added HRC, from February 2020 to August 2021 at Kumamoto University Hospital, were used as the training and validation data sets. These 14 patients included eight men (mean age, 40.4 years; range, 15–82 years) and six women (mean age, 43.8 years; range, 21–73 years). No artifact was found on any of the images.

 The data set for each final test of the three methods, indirect, direct, and direct+HRC, consisted of 13 patients (6 men: mean age, 58.7 years; range, 31-82 years; and 7 women: mean age, 54.6 years; range, 33-75 years) who underwent all three imaging modalities, including head MRI and PET/CT.

***Image acquisition***

^18^F-FDG brain PET/CT imaging was performed using the Vereos PET/CT system (Philips Medical Systems, Amsterdam, Netherlands), and the MRI was performed using the MAGNETOM Prisma 3.0T system (Siemens Healthcare, Erlangen, Germany), under the same conditions. ^18^F-FDG PET acquisition conditions were as follows: matrix size, 128×128; pixel size, 2 mm; acquisition time, 10 min; acquisition mode, three-dimensional list mode; reconstruction method, three-dimensional ordered subsets expectation maximization (3D blob-based OSEM); iteration, 3; and subset number, 15 (17). The HRC images were obtained by using the image deconvolution by the Richardson Lucy algorithm (18-20). Before PET imaging, CT was performed at 120 kV, 49 mAs, scan time 3.5 sec, FOV600, matrix size, 512×512 (voxel size 1.17×1.17×2.00 mm^3^) for CT attenuation correction (CTAC) of PET images using the PET/CT system. The MRI sequence consisted of magnetization and rapid acquisition with gradient echo using magnetization-prepared rapid acquisition with the gradient echo (MPRAGE; three-dimensionalT1WI) method at FOV 240mm ×240mm, matrix：480×480, voxel size 0.5×0.5×1.00 mm^3 ^(21,22).

***Image conversion and fusion***

**Figure 1:**
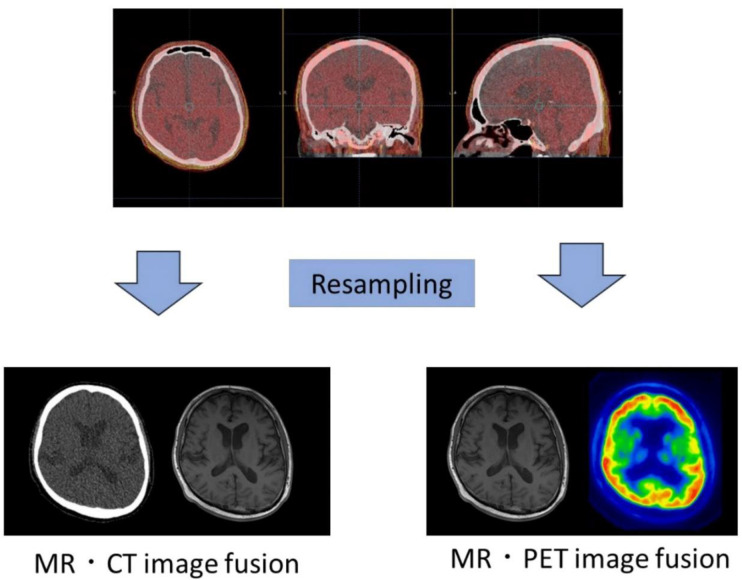
Image conversion and fusion. MRI, CT, and PET images were converted such that the matrix size and pixel size of the images were fused with each other. These images were reconstructed such that the anatomical positions coincided with each other.

 For each participant, pairs of cranial MRI, CT, and PET images were superimposed correctly using Mirada DBx Build 1.1.1.3 (64 bit; Mirada Medical, Ltd., Oxford, UK) (Figure 1). The PET and CT images were resized to match the dimensions of the MRI images and these were then integrated with one another. These images were reconstructed such that the anatomical landmarks coincided with each other.

 For CT images, the window width and window level were arbitrarily set at 2,500 and 250, respectively, for best depiction of the bones. Then, areas other than the head (e.g. the bed) were cropped from the images.

 With MRI, heterogeneity of the magnetic field owing to differences in the device and patient physique can lead to heterogeneity of signal intensity; therefore, the maximum signal intensity is standardized as 255. Additionally, the region other than the head was cropped out of the figures. PET images showed differences between the participants in the image pixel counts and, thus, it was necessary to make the count scale consistent for each participant and to align the counts with the formula used in Equation 1 (shown below). With each pixel, the minimum count for the relevant participant was subtracted, and the result was divided by the difference between the maximum and minimum counts to obtain the normalized value:

X_norm_= ((X-X_min_)/(X_max_-X_min_))×255 (1)

X_norm_: normalized pixel value; 

X_min_: minimum value for the participant;

X_max_: maximum value for the participant

 The image data for each patient was individually normalized using this equation.

***Image format transformation***

 The image format required for DCNN processing is the Portable Network Graphics (PNG) format. Therefore, after image conversion and fusion, the PET, CT, and MRI images in the Digital Imaging and Communications in Medicine (DICOM) format were converted to the PNG format using our original program developed in the Python programming language.

 All MRI, CT, and PET images were processed as detailed above and, then, prepared as 256×256 matrices. Additionally, by performing four-way rotation, four-way translation, and flipping, the number of images was expanded 32-fold, and the learning data set was prepared. For the final image, the PNG image obtained using DCNN was reformatted into a DICOM image using the original image pixel counts.

***Synthetic CT (SCT) image generation using a DCNN***

 Using a DCNN, AC images were generated by the indirect, direct, and direct+HRC methods. 

**Figure 2:**
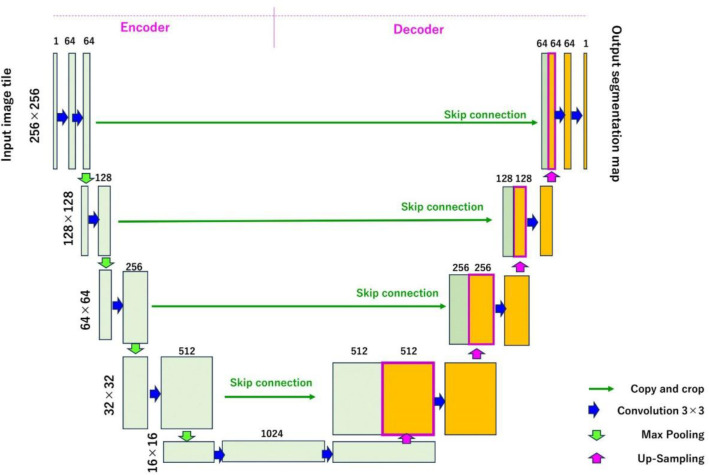
Structure of the U-net

Figure 2 shows the U-net structure of the DCNN used to generate the SCT images (23-25). In a U-net of 13 convolutional layers in five depth grades, the activation coefficient (rectified linear unit) and batch normalization were applied to all convolutional layers other than the last one. Immediately after the last convolutional layer, the squared error was applied as the output layer. Using a method for a stochastic optimization program (Adam, version 9) (24), taking the optimization coefficients for U-net learning to be 0.001, 0.9, 0.999, and 10-8 for α, β-1, β-2, and ε, respectively. For the use environment, the operating system was Windows 10 Pro (Microsoft Corporation, Redmond, WA, USA), the central processing unit was Intel(R) Xeon(R) Gold 5218R CPU (Intel Corporation, Santa Clara, CA, USA), and the graphics processing unit was NVIDEA Quadro RTX6000 (NVIDIA Corporation, Santa Clara, CA, USA). For the development environment, the Neural Network Console (Sony Corporation, Tokyo, Japan) was used.

***Indirect method***

**Figure 3:**
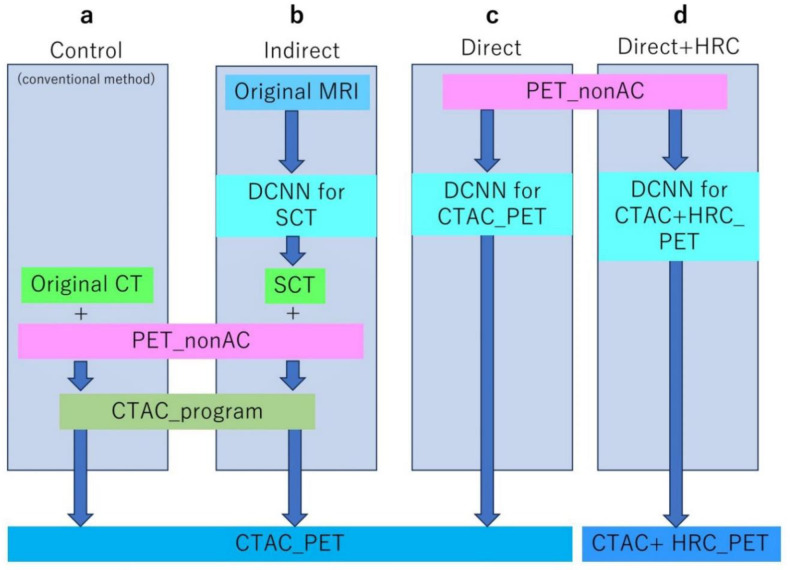
Flow diagram of the indirect, direct and direct+HRC method

 Taking pairs of MRI and CT images with which expansion processing had been performed as input images, learning and validation were performed using the DCNN (Figure 3b). Using only the original image, with which no expansion processing had been performed, the SCT image corresponding to the real MRI image was generated. The number of learning repetitions in U-net was 50, and the learning curve was converged at this time. U-net learning and validation involved random allocation of all samples to six groups and evaluation of all participants through six-fold cross-validation. 

SCT and non-AC images were input into AC_CT attenuation correction software (PDRadiophama Inc.) and, thus, AC images were generated. The software-created μ-map of the SCT image was defined by the HU values of the CT: air (below 200 HU), bone (above 250 HU), and soft tissue (between 200 and 250 HU).

***Direct method***

 With pairs of expansion-processed non-AC and original CTAC PET images as input images, learning and validation were performed with the DCNN. Using only the original non-AC image, with which no expansion processing had been performed, the CTAC image corresponding to the real non-AC image was generated (Figure 3c). The number of times learning in U-net was repeated was 30, and the learning curve was converged at this time. For U-net learning and validation, all samples were randomly allocated to seven groups, and evaluation of all participants was performed by seven-fold cross-validation.

***Direct+HRC method***

 With pairs of expansion-processed non-AC and original CTAC+HRC images as input images, learning and validation were performed with the DCNN (Figure 3d). Using only the original image, with which no expansion processing had been performed, an AC image corresponding to the direct HRC image was generated. The learning in U-net was repeated 30 times, and the learning curve was converged at this time. For U-net learning and validation, all samples were randomly allocated to 10 groups, and evaluation of all participants was performed through 10-fold cross-validation.

***Evaluation***

 AC images obtained by the indirect, direct and direct+HRC methods were compared with original CTAC PET images and original CTAC+HRC PET images by using the evaluated using normalized mean square error (NMSE）peak-signal-to-noise ratio (PSNR) and structural similarity (SSIM), respectively. 

 With the output images, using the image-processing software (Daemon Research Image Processor (DRIP) version 3.0.2.0; PDRadiophama Inc, Tokyo, Japan), the normalized mean square errors (NMSEs) were calculated. The NMSE calculation formula was as shown in Equation 2:

NMSE=∑i=1x∑j=1y[Xi，j-Yi，j]2∑i=1x∑j=1y Y(i，j)² (2) 

i: matrix size (x direction), 

j: matrix size (y direction), 

X: reference image, 

Y: target image (normalized to the maximum value of the reference image).

 The NMSEs were expressed as mean square errors of the evaluated images and reference images, and the difference between the two types of images was presented. The closer the NMSE approximated to 0, the closer the image was to the reference image. Moreover, the structural similarity (SSIM) of the output images was calculated using a program prepared with Python; the calculating formulas are presented in Equation 3, 4(26).

$\mathrm{PSNR}=10\log_{10}(\frac{\max(K)}{{\left\|K-\mathrm{K'}\right\|}_{2}^{2}})$ (3)

Where K and K’ represent the ground truth (the original image) and the indirect, direct, and direct+HRC images, respectively.

$\mathrm{SSIM}=\frac{\left(2\mu x\mu y+c1\right)\left(2\sigma \mathrm{xy}+c2\right)}{\left({\mu x}^{2}+{\mu y}^{2}+c1\right)\left({\sigma x}^{2}+{\sigma y}^{2}+c2\right)}$ (4)

μx: the pixel sample mean of x, 

μy: the pixel sample mean of y, 

σx^2^: the variance of x, 

σy^2^: the variance of y, 

σxy: the covariance of x and y, 

c1= (k1L)^2^, 

c2= (k2L)^2^; 

k1=0.01 

k2=0.03 constants, 

L is the dynamic range of pixel values.

 In an evaluation method involving SSIM, the correlations of the surrounding pixel enabled changes in brightness, contrast, and structure should be considered. The closer the SSIM was to 1, the smaller was the error in relation to the reference image.

 The values of the indirect and direct methods were compared with the value of the original CTAC method, respectively. The values of the direct+HRC method were compared with the original CTAC+HRC method.

***Statistical analyses***

 Statistical analyses were performed using EZR (Saitama Medical Center, Jichi Medical University, Saitama, Japan) version 1.54 (27), and the graphical user interface of R (The R Foundation for Statistical Computing, Vienna, Austria) version 3.6.2. The NMSE, PSNR and SSIM values of the three methods were compared using the Wilcoxon rank sum test. Statistical significance was defined as P<0.05.

## Results

**Figure 4:**
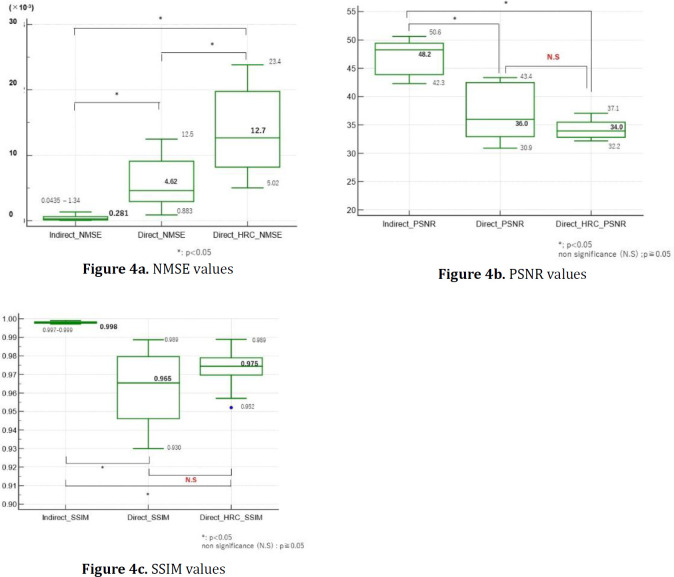
Box-and-whisker plots generated using the Wilcoxon rank sum test

Figure 4a shows box-and-whisker plots generated using the Wilcoxon rank sum test for NMSE values obtained through the DCNN and original methods. The NMSE values for the indirect method were distributed from 0.0435×10^-3^ to 1.34×10^-3^ (median 0.281×10^-3^). 

 For the direct method, the values were distributed from 0.833×10^-3^ to 12.5×10^-3^ (median 4.62×10^-3^), while for the direct+HRC method, they were distributed from 5.02×10^-3^ to 23.4×10^-3^ (median 12.7×10^-3^). The significance levels for each method were all p<0.05, indicating statistical significance. Additionally, there was a noticeable difference in the median values among the three groups. The NMSE median increased in the following order: indirect > direct > direct+HRC, and the range from the lowest to the highest values also expanded in the same manner.

Figure 4b shows box-and-whisker plots of PSNR values obtained by the DCNN and original methods. The PSNR values for the indirect method was 47.0±2.88 (median 48.2). For the direct method, the value was 37.0±4.71 (median 36.0), and for the direct+HRC method, it was 35.0±3.80 (median 34.0). The significance levels between the indirect and direct methods were all p<0.05, and between the indirect and direct+HRC methods, p<0.05 as well. There was a noticeable difference in the median values among these groups. However, the significance level between the direct and direct+HRC methods was p>0.05, indicating no statistically significant difference in the rank means of the groups. The PSNR decreased in the following order: indirect > direct > direct+HRC, and the range of values also shrink accordingly.

Figure 4c presents box-and-whisker plots of SSIM values obtained by the DCNN and original methods. The SSIM value for the indirect method was 0.998±0.00016 (median 0.998). 

 For the direct method, the value was 0.961±0.0058 (median 0.965), and for the direct+HRC method, it was 0.975±0.0021 (median 0.975). The significance levels were all p<0.05 when comparing the indirect and direct methods, as well as between the indirect and direct+HRC methods. There was a noticeable difference in the median values among these groups. However, the significance level between the direct and direct+HRC methods was p>0.05, indicating no statistically significant difference in the rank means of the groups. The SSIM median decreased in the following order: indirect > direct+HRC > direct, and the range of values also expanded accordingly.

**Figure 5:**
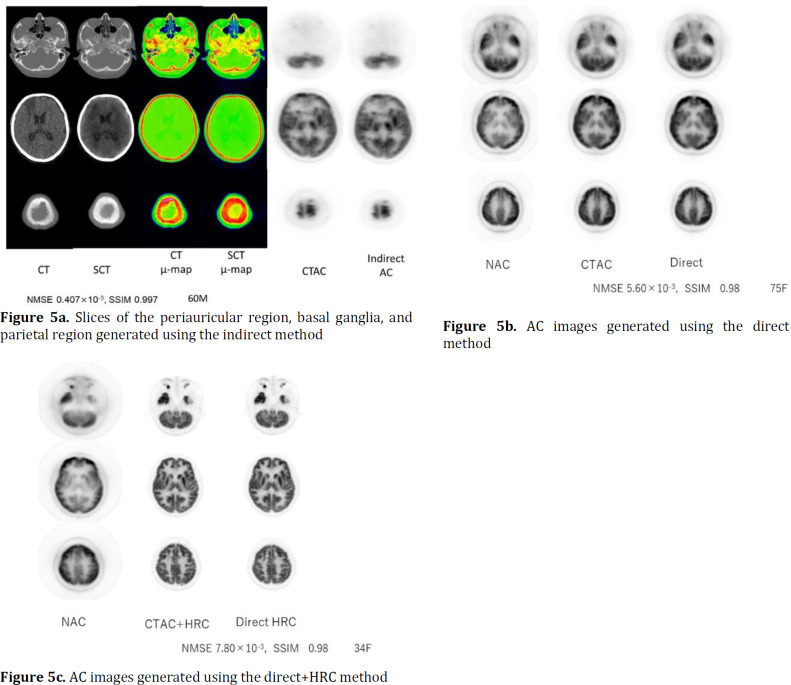
Clinical images

Figure 5a-c show clinical images of the indirect, direct and direct+HRC methods, respectively. Figure 5a shows slices of the periauricular region, basal ganglia, and parietal region are presented. On the left, there are CT, SCT, CT-µ-map, and SCT-µ-map images, along with AC images that have undergone attenuation correction using both the CTAC and indirect methods. The NMSE and SSIM values for the AC images obtained with the CTAC and indirect methods were 0.407×10^-3^ and 0.997, respectively. The images were generated with recognition of even minor differences in CT values, and no differences between the CT images were found. In the μ-map images, some differences in bone structure were shown in the slices of the parietal region. Since the AC images of the parietal region were generated by using the mean μ values of the same area, the differences of the μ-map images did not affect AC imaging.

Figure 5b shows the AC image generated by the direct method for one subject. The NMSE and SSIM of this participant’s AC images obtained using the CTAC and direct methods were 5.60×10^-3^ and 0.98, respectively. Above, slices of the periauricular region, basal ganglia, and parietal region are presented; on the left, non-AC and AC images with attenuation correction by CTAC and direct methods are presented. The inaccuracy of the pixels predicted using the DCNN were not directly reflected in the PET images.

Figure 5c shows the AC image generated using the direct+HRC method for one subject. The NMSE and SSIM of this participant’s AC images obtained using the CTAC and direct methods were 7.80×10^-3^ and 0.98, respectively. Slight differences in coloration intensity were shown in the corpus striatum and brain parenchyma. It was possible to generate AC images presenting patients’ individual anatomical characteristics even in slices from the periauricular region, basal ganglia, and parietal region. Based on the high resolution of the AC images obtained using CTAC, which were the teaching images, and the clarity at the border region of the tissue, it was possible to clearly define the information in each of the pixels.

 Visual evaluation revealed no difference between AC images. All generation of AC images were very good, and no major changes of image counts or lack of clarity were detected.

 Visual evaluation revealed no difference between AC images obtained by the CTAC method and images obtained by the indirect, direct, and direct+HRC methods.

## Discussion

 In this study, the following attenuation correction methods were developed: (i) the indirect method, in which attenuation correction of cranial PET is performed using CT images predicted from cranial MRI images by a DCNN; (ii) the direct method, in which AC images are generated directly from non-AC images; and (iii) the direct+HRC method, in which resolution correction is performed up to a high level simultaneously with the direct method.

 The NMSE and SSIM obtained by each of these methods were compared (Figure 4a and 4c). The NMSE value increased in the order of indirect, direct, and direct+HRC methods, which were 0.281×10^-3^, 4.62×10^-3^, and 12.7×10^-3^, respectively (Figure 4a). The difference between the indirect and the direct and direct+HRC values was approximately 10-fold. 

 Dong. et al. reported that the NMSE using an encoder-decoder-based deep learning approach in the indirect method was 7.00×10^-3^. 

 The direct value of 4.62×10^-3^, obtained for our direct method, was close to Dong et al.'s value of 7.00×10^-3^ (55data) (12). However, our direct+HRC NMSE value of 12.7×10^-3^ was 50% inferior to Dong et al.'s value of 7.00×10^-3^ using a direct method. The PSNR value decreased in the order of indirect, direct methods and direct+HRC, which were 47.0, 37.0 and 35.0 (Figure 4b). The SSIM value decreased in the order of indirect, direct+HRC, and direct methods, which were 0.998, 0.975, and 0.961, respectively (Figure 4c). The difference in PSNR and SSIM exhibited a similar trend in all three methods. These results support the results of the evaluation using SSIM (28). Arabi et al. reported that the SSIM value using an indirect method was 0.93 (40 data learning) (13). Shiri et al. reported that the SSIM value using a direct method (deep encoder-decoder) was 0.989 (129 data learning) (11). Arabi et al. reported that the SSIM value using the other direct method was 0.94 (180 data) (29). Additionally, 

a meta-analysis by Raymond et al. reported a SSIM value of 0.95 in four papers on attenuation correction using deep learning, and this result is no different from the SSIM values of the direct+HRC method (28). Therefore, all generations of AC images from the indirect, direct, and direct+HRC methods were of excellent quality because they exceeded the SSIM values of previous reports (10-13).

 The PSNR and SSIM of the direct+HRC method were approximately equivalent to the direct method (p=0.414 (Figure 4b, p=0.11 (Figure 4c)). However, the range of the values in the direct+HRC method was narrower than that of the direct method. The algorithm used for HRC uses high resolution and regularization noise suppression techniques (30). The direct+HRC images has been trained on the original denoised higher resolution images compared to the direct images. The DCNN is able to learn finer features (complexity) between adjacent pixels during training more effectively than in the case of the direct images. Consequently, the DCNN output can represent finer structures compared to the direct images, owing to the high-resolution training. As a result, SSIM was higher for the direct+HRC compared to the direct method because SSIM considered brightness, contrast, and structure in images. On the other hand, NMSE considered the absolute value of errors, so the results are reversed due to the impact of noise and other factors when representing fine structures.

 Visual evaluation revealed no difference between AC images, and no major changes of image counts or lack of clarity were detected. 

 Based on the original HRC+AC images obtained using CTAC, which were the teaching images, and the clarity at the border region of the tissue, it was possible to clearly define the information in each of the pixels. This method is as reliable as the previously reported attenuation correction method. In addition, this method has resolution correction, which is expected to provide fine structural information on the striatum and other areas in the brain.

 Overall, based on the provided information, it appears that the deep learning-based attenuation correction method is not only reliable, with image quality comparable to traditional methods but also offers additional benefits, particularly in terms of resolution correction for detailed brain structure information.

 Our study highlights a significant advantage of the direct+HRC method. We included resolution correction, which is expected to provide fine structural information in the striatum and other areas of the brain. With the direct+HRC method, input of non-AC images enabled image generation with resolution- and attenuation-correction within seconds. These factors are expected to reduce human error and personal load in clinical practice due to less time requirements for radiography and image-processing.

 Sun et al. reported high-quality PET images generated from ultra-low-dose PET/MRI using bi-task deep learning. The NMSE values of the method were distributed from 2.42×10^-3^ to 3.09×10^-3^, and the SSIM values of this method were over 0.98 (24).

 Therefore, for clinical use, it is essential to ensure that the image quality of our direct+HRC method is consistent with these values. In this study, we successfully created high-resolution images with only 14 training examples. It is highly likely that with a substantial increase in the amount of training data, there is a significant potential for improvement in NMSE and SSIM values. Furthermore, it's important to note that this study is based on results from a single facility and device. Therefore, further validation across multiple facilities and devices will be necessary in the future.

 As a new technology using deep learning, attention has been paid to generative adversarial networks (GANs). A GAN is constructed from two networks, the generator and discriminator, and their mutual competition generates images with high resolution (31). Therefore, it is expected that precision can be further increased by switching the network structures used for image generation from U-net to GAN.

## Conclusion

 A DCNN-based method for direct attenuation and high-resolution correction has promising results and potential for clinical use, aligning closely with previous methods. To enhance its applicability, it is crucial to increase training instances with data from facilities with diverse characteristics. Collaboration among institutions and wider validation across various scenarios are needed to ensure its effectiveness in real-world medical practice.

## Data Availability

The datasets generated and/or analyzed during the current study are available from the corresponding author upon reasonable request.
